# State Cannabis Legalization and Trends in Cannabis-Related Disorders in US Older Adults, 2017 to 2022

**DOI:** 10.1001/jamanetworkopen.2024.17634

**Published:** 2024-06-18

**Authors:** Silvia Perez-Vilar, Pablo Freyria Duenas, Rose Radin, Sandia Akhtar, Michael Wernecke, Jeffrey A. Kelman, David J. Graham

**Affiliations:** 1 Center for Drug Evaluation and Research, US Food and Drug Administration, Silver Spring, Maryland; 2 Acumen LLC, Burlingame, California; 3 Centers for Medicare & Medicaid Services, Washington, DC

## Abstract

This cross-sectional study examines trends in health care encounters with cannabis-related disorders among Medicare beneficiaries from 2017 to 2022.

## Introduction

An increasing number of US states and territories have enacted laws allowing adult and/or medical use of marijuana (cannabis).^1^ As expanded access coincides with increased prevalence of medical and nonmedical cannabis use,^2^ we characterized trends in health care encounters with cannabis-related disorders among Medicare beneficiaries aged 65 years or older by state or territory cannabis legal status.

## Methods

This cross-sectional study qualified as public health surveillance and was exempt from review by the US Food and Drug Administration institutional review board. Informed consent was not required because data were deidentified. The study followed the STROBE reporting guideline.

We used Medicare enrollment records, Fee-for-Service (FFS) claims, and Medicare Advantage (MA) encounter data yearly, from 2017 to 2022 to conduct a descriptive study among beneficiaries aged 65 years or older continuously enrolled in FFS (Parts A, B) or MA (Parts A, B, C) for 183 days or more before the first day of the calendar year (index date) and through the full calendar year or death.^3,4^ We excluded long-term nursing home residents and beneficiaries receiving dialysis. We identified health care encounters with cannabis-related disorders using the *ICD-10-CM *diagnosis codes (F12x). We conducted all analyses by cannabis legal status (illegal, medical, and adult and medical) in the beneficiaries’ state or territory of residence, as established by law at the beginning of each calendar year (eTable in Supplement 1). We further stratified by encounter claim setting and by Medicare type. We computed annual encounter rates per 10 000 beneficiaries with 95% CIs, estimated the mean annual change in rates, and assessed statistical significance using Mann-Kendall tests using SAS version 9.4 (SAS Institute) and R version 4.1.2 (R Project for Statistical Computing). Statistical significance was set at *P* < .05. Data were analyzed from August to December 2023.

## Results

**Figure 1.**
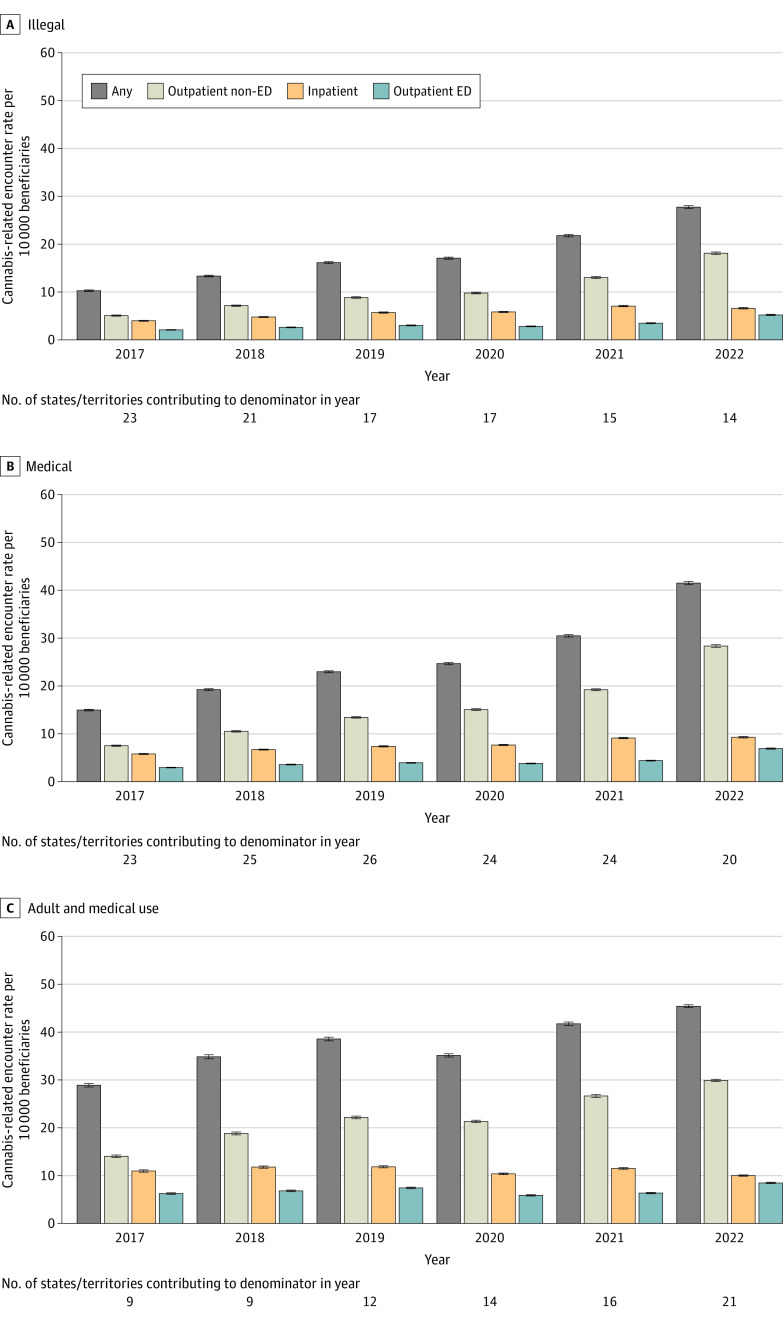
Annual Rates of Cannabis-Related Disorder Encounters by State or Territory Cannabis Legal Status, Medicare, 2017-2022 A, In states where cannabis was illegal, the Mann-Kendall statistic for any setting was 1.0; outpatient non-ED, 1.0; inpatient, 0.9; outpatient ED, 0.9. The linear model slope for any setting was 3.2 (95% CI, 2.5-4.0); outpatient non-ED, 2.4 (95% CI, 1.7-3.0); inpatient, 0.6 (95% CI, 0.4-0.8); outpatient ED, 0.5 (95% CI, 0.3-0.8). B, In states where cannabis was legal for medical use, the Mann-Kendall statistic for any setting was 1.0; outpatient non-ED, 1.0; inpatient, 1.0; outpatient ED, 0.9. The linear model slope for any setting was 4.8 (95% CI, 3.4-6.2); outpatient non-ED, 3.8 (95% CI, 2.6-4.9); inpatient, 0.7 (95% CI, 0.6-0.8); outpatient ED, 0.6 (95% CI, 0.3-1.0). C, In states where cannabis was legal for adult and medical use, the Mann-Kendall statistic for any setting was 0.9; outpatient non-ED, 0.9; inpatient, −0.3; outpatient ED, 0.3. The linear model slope for any setting was 2.9 (95% CI, 1.7-4.0); outpatient non-ED, 2.9 (95% CI, 2.2-3.6); inpatient, −0.2 (95% CI, −0.6 to 0.1); outpatient ED, 0.2 (95% CI, −0.2 to 0.7). ED indicates emergency department.

**Figure 2.**
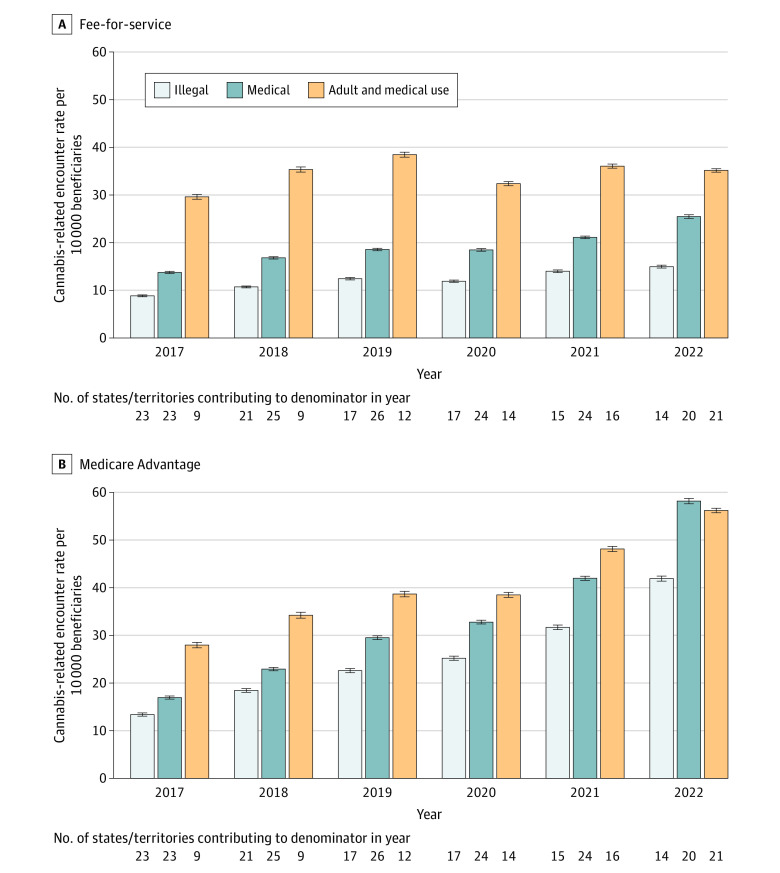
Annual Rates of Cannabis-Related Disorder Encounters by Medicare Type and State or Territory Cannabis Legal Status, 2017-2022 A, For fee-for-service Medicare, the Mann-Kendall statistic in states where cannabis use was illegal was 0.9; legal for medical use, 0.9; and legal for adult and medical use, 0.2. The linear model slope for states where cannabis was illegal was 1.1 (95% CI, 0.8 to 1.4); legal for medical use, 2.0 (95% CI, 1.5 to 2.6); legal for adult and medical use, 0.7 (95% CI, −0.8 to 2.2). B, For Medicare Advantage, the Mann-Kendall statistic in states where cannabis use was illegal was 1.0; legal for medical use, 1.0; and legal for adult and medical use, 0.9. The linear model slope for states where cannabis was illegal was 5.3 (95% CI, 4.1 to 6.4); legal for medical use, 7.6 (95% CI, 5.7 to 9.5); legal for adult and medical use, 5.2 (95% CI, 3.9 to 6.5).

The eligible population included 55 941 880 unique beneficiaries during the study. Rates of health care encounters with cannabis-related disorders increased from 2017 through 2022, irrespective of state or territory cannabis legal status (Figure 1). Rates were greatest in states or territories with both adult and medical use legalization (45.4 [95% CI, 45.1-45.7] per 10 000 beneficiaries in 2022), followed by states or territories with medical legalization (41.5 [95% CI, 41.2-41.8] per 10 000 beneficiaries in 2022), and states or territories where cannabis use was illegal (27.7 [95% CI, 27.4-28.0] per 10 000 beneficiaries in 2022). We noted the greatest increasing trends in nonemergency department (ED) outpatient settings across all legalization categories (Figure 1). We observed higher average annual increases among beneficiaries enrolled in MA than those enrolled in FFS (Figure 2).

## Discussion

Rates of cannabis-related disorder encounters increased from 2017 through 2022 among US Medicare-insured older adults. We observed the highest rates in states or territories that legalized adult and medical use of cannabis. Our results also suggest higher average annual increases in states or territories that legalized medical cannabis. The observed increases in rates were driven by non-ED outpatient encounters irrespective of the cannabis legal status. We observed larger upward trends in rates among MA enrollees than among those in FFS. Overall, data suggest that increasing rates of health care encounters documenting cannabis-related disorders among older adults might be associated with the type of cannabis legalization. However, differences in cannabis use patterns and perception of risk may influence policy changes and present challenges to causal inference.

Additional limitations include the descriptive nature of this study, where other factors that may affect results were not accounted for, such as demographics, socioeconomic indicators, comorbidities, the COVID-19 pandemic, inability to distinguish between marijuana and federally legal hemp,^5^ and differences in policies between states or territories with the same cannabis legal status. Differences in legality may lead to differences in the disclosure of cannabis use as well as in the detection, diagnosis, and recording of cannabis-related disorders. Differences between FFS and MA billing incentives, out-of-pocket spending, and tools available to manage beneficiaries’ care could lead to differences in how clinicians use the *ICD-10-CM *codes we relied on.^6^

## Notes

Supplement 1. **eTable.** Cannabis Legal Status Categorization by State or Territory and Year

Supplement 2.
Data Sharing Statement
