# Supplementary material for: State Cannabis Legalization and Trends in Cannabis-Related Disorders in US Older Adults, 2017 to 2022
Source: JAMA Netw Open. 2024 Jun 18;7(6):e2417634. doi: 10.1001/jamanetworkopen.2024.17634 (PMC11185962; doi:10.1001/jamanetworkopen.2024.17634)
Supplement: Supplement 1. — eTable. Cannabis Legal Status Categorization by State or Territory and Year [file jamanetwopen-e2417634-s001.pdf]

Supplemental Online Content

Perez-Vilar S, Freyria Duenas P, Radin R, et al. State cannabis legalization and trends in cannabis-related disorders in US older adults, 2017 to 2022. *JAMA Netw Open*. 7(6):e2417310. doi:10.1001/jamanetworkopen.2024.17310

eTable. Cannabis Legal Status Categorization by State or Territory and Year

This supplemental material has been provided by the authors to give readers additional information about their work.

© 2024 Perez-Vilar S et al. *JAMA Network Open.*

eTable 1. Cannabis Legal Status Categorization by State/Territory and Year

State/ Territory Name	2017	2018	2019	2020	2021	2022
**Alabama**	Illegal	Illegal	Illegal	Illegal	Illegal	Medical
**Alaska**	Medical & Adult use	Medical & Adult use	Medical & Adult use	Medical & Adult use	Medical & Adult use	Medical & Adult use
**Arizona**	Medical	Medical	Medical	Medical	Medical & Adult use	Medical & Adult use
**Arkansas**	Medical	Medical	Medical	Medical	Medical	Medical
**California**	Medical & Adult use	Medical & Adult use	Medical & Adult use	Medical & Adult use	Medical & Adult use	Medical & Adult use
**Colorado**	Medical & Adult use	Medical & Adult use	Medical & Adult use	Medical & Adult use	Medical & Adult use	Medical & Adult use
**Connecticut**	Medical	Medical	Medical	Medical	Medical	Medical & Adult use
**Delaware**	Medical	Medical	Medical	Medical	Medical	Medical
**District of Columbia**	Medical & Adult use	Medical & Adult use	Medical & Adult use	Medical & Adult use	Medical & Adult use	Medical & Adult use
**Florida**	Medical	Medical	Medical	Medical	Medical	Medical
**Georgia**	Illegal	Illegal	Illegal	Illegal	Illegal	Illegal
**Hawaii**	Medical	Medical	Medical	Medical	Medical	Medical
**Idaho**	Illegal	Illegal	Illegal	Illegal	Illegal	Illegal
**Illinois**	Medical	Medical	Medical	Medical & Adult use	Medical & Adult use	Medical & Adult use
**Indiana**	Illegal	Illegal	Illegal	Illegal	Illegal	Illegal
**Iowa**	Illegal	Medical	Medical	Medical	Medical	Medical
**Kansas**	Illegal	Illegal	Illegal	Illegal	Illegal	Illegal
**Kentucky**	Illegal	Illegal	Illegal	Illegal	Illegal	Illegal
**Louisiana**	Medical	Medical	Medical	Medical	Medical	Medical
**Maine**	Medical & Adult use	Medical & Adult use	Medical & Adult use	Medical & Adult use	Medical & Adult use	Medical & Adult use
**Maryland**	Medical	Medical	Medical	Medical	Medical	Medical
**Massachusetts**	Medical & Adult use	Medical & Adult use	Medical & Adult use	Medical & Adult use	Medical & Adult use	Medical & Adult use
**Michigan**	Medical	Medical	Medical & Adult use	Medical & Adult use	Medical & Adult use	Medical & Adult use
**Minnesota**	Medical	Medical	Medical	Medical	Medical	Medical
**Mississippi**	Illegal	Illegal	Illegal	Illegal	Illegal	Illegal
**Missouri**	Illegal	Illegal	Medical	Medical	Medical	Medical
**Montana**	Medical	Medical	Medical	Medical	Medical & Adult use	Medical & Adult use

© 2024 Perez-Vilar S et al. *JAMA Network Open.*

**Nebraska**	Illegal	Illegal	Illegal	Illegal	Illegal	Illegal
**Nevada**	Medical & Adult use	Medical & Adult use	Medical & Adult use	Medical & Adult use	Medical & Adult use	Medical & Adult use
**New Hampshire**	Medical	Medical	Medical	Medical	Medical	Medical
**New Jersey**	Medical	Medical	Medical	Medical	Medical	Medical & Adult use
**New Mexico**	Medical	Medical	Medical	Medical	Medical	Medical & Adult use
**New York**	Medical	Medical	Medical	Medical	Medical	Medical & Adult use
**North Carolina**	Illegal	Illegal	Illegal	Illegal	Illegal	Illegal
**North Dakota**	Medical	Medical	Medical	Medical	Medical	Medical
**Ohio**	Medical	Medical	Medical	Medical	Medical	Medical
**Oklahoma**	Illegal	Illegal	Medical	Medical	Medical	Medical
**Oregon**	Medical & Adult use	Medical & Adult use	Medical & Adult use	Medical & Adult use	Medical & Adult use	Medical & Adult use
**Pennsylvania**	Medical	Medical	Medical	Medical	Medical	Medical
**Rhode Island**	Medical	Medical	Medical	Medical	Medical	Medical
**South Carolina**	Illegal	Illegal	Illegal	Illegal	Illegal	Illegal
**South Dakota**	Illegal	Illegal	Illegal	Illegal	Medical	Medical
**Tennessee**	Illegal	Illegal	Illegal	Illegal	Illegal	Illegal
**Texas**	Illegal	Illegal	Illegal	Illegal	Illegal	Illegal
**Utah**	Illegal	Illegal	Medical	Medical	Medical	Medical
**Vermont**	Medical	Medical	Medical & Adult use	Medical & Adult use	Medical & Adult use	Medical & Adult use
**Virginia**	Illegal	Illegal	Illegal	Illegal	Medical	Medical & Adult use
**Washington**	Medical & Adult use	Medical & Adult use	Medical & Adult use	Medical & Adult use	Medical & Adult use	Medical & Adult use
**West Virginia**	Illegal	Medical	Medical	Medical	Medical	Medical
**Wisconsin**	Illegal	Illegal	Illegal	Illegal	Illegal	Illegal
**Wyoming**	Illegal	Illegal	Illegal	Illegal	Illegal	Illegal
**American Samoa**	Illegal	Illegal	Illegal	Illegal	Illegal	Illegal
**Guam**	Medical	Medical	Medical	Medical & Adult use	Medical & Adult use	Medical & Adult use
**Northern Marianas**	Illegal	Illegal	Medical & Adult use	Medical & Adult use	Medical & Adult use	Medical & Adult use
**Other U.S. Possessions***	Medical	Medical	Medical	Medical	Medical	Medical

Primary Source: State Medical Cannabis Laws. National Conference of State Legislatures. https://www.ncsl.org/health/state-medical-cannabis-laws.

Cannabis legal status was determined based on the latest cannabis law enacted (i.e., the law is agreed upon and made official) as of the first day of each calendar year.

**U.S possessions legality status categorization defined by status in Puerto Rico, the largest territory by population among the "U.S. Possessions" considered.*

© 2024 Perez-Vilar S et al. *JAMA Network Open.*
