# Supplementary material for: State Cannabis Legalization and Trends in Cannabis-Related Disorders in US Older Adults, 2017 to 2022
Source: JAMA Netw Open. 2024 Jun 18;7(6):e2417634. doi: 10.1001/jamanetworkopen.2024.17634 (PMC11185962; doi:10.1001/jamanetworkopen.2024.17634)
Supplement: Supplement 2. — Data Sharing Statement [file jamanetwopen-e2417634-s002.pdf]

Data Sharing Statement

Perez-Vilar. State Cannabis Legalization and Trends in Cannabis-Related Disorders in US Older Adults, 2017 to 2022. *JAMA Netw Open*. Published June 18, 2024. doi:10.1001/jamanetworkopen.2024.17634

Data
Data available: No

Additional Information
Explanation for why data not available: Data will not be shared in order to protect patient confidentiality.
